# 40 years of Alma Ata Malaysia: targeting equitable access through organisational and physical adaptations in the delivery of public sector primary care

**DOI:** 10.1017/S146342362000002X

**Published:** 2020-02-24

**Authors:** Fariza Fadzil, Safurah Jaafar, Rohana Ismail

**Affiliations:** 1 Master of Public Health, Senior Principal Assistant Director, Primary Health Infrastructure Development Sector, Primary Healthcare Section Family Health Development Division, Ministry of Health Malaysia, Putrajaya, Malaysia; 2 Master of Public Health, Master of Business Administration, Former Director Family Health Development Division, Ministry of Health Malaysia, Currently Professor Community Medicine, International Medical University Malaysia, Kuala Lumpur, Malaysia

**Keywords:** access, equity, PHC infrastructure, universal health coverage, urban–rural divide

## Abstract

This paper illustrates the development of Primary Health Care (PHC) public sector in Malaysia, through a series of health reforms in addressing equitable access. Malaysia was a signatory to the Alma Ata Declaration in 1978. The opportunity provided the impetus to expand the Rural Health Services of the 1960s, guided by the principles of PHC which attempts to address the urban–rural divide to improve equity and accessibility. The review was made through several collation of literature searches from published and unpublished research papers, the Ministry of Health annual reports, the 5-year Malaysia Plans, National Statistics Department, on health systems programme and infrastructure developments in Malaysia. The Public Primary Care Health System has evolved progressively through five phases of organisational reforms and physical restructuring. It responded to growing needs over a 40-year period since the Alma Ata Declaration in 1978, keeping equity, accessibility, efficiency and universal health coverage consistently in the backdrop. There were improvements of maternal, infant mortality rates as well as accessibility to health services for the population. The PHC Reforms in Malaysia are the result of structured and strategic investment. However, there will be continuing dilemma between cost-effectiveness and equity. Hence, continuous efforts are required to look at opportunity costs of alternative strategies to provide the best available solution given the available resources and capacities. While recognising that health systems development is complex with several layers and influencing factors, this paper focuses on a small but crucial aspect that occupies much time and energies of front-line managers in the health.

## Background

Equity and access through Primary Health Care (PHC) have been the main agenda of many countries after the PHC Declaration made in Kazakhstan in 1978. Each country including Malaysia used unique approaches to address this mission, based on its resources and presiding policies. Since independence in 1957, Malaysia has regarded health system development as an integral component of national development as evidenced in successive Malaysia Development Plans (Perdana Leadership Foundation, 2018). In earlier years, the focus was on reducing the urban–rural divide, while more recently the focus is on equity, quality, efficiency and universal health coverage. Development of physical infrastructure such as transport and communication, educational and health facilities have provided the platform to support the provision and use of health-care services that in turn have been influenced by the interplay of resources including equipment, logistics, transport, supplies, manpower, management and information.

Health care requires the establishment of a comprehensive variety of buildings, to cater for different functions that are needed during the life span of man ‘from womb to tomb’ (Mohd Nawawi et al., 2012). It requires appropriate best match between requirements of space, patient and information flow and clinic services to function in harmony. Each facility would need to fit with the social development of the local area, and not be under- or over-utilised during a specified period.

## Methods

The paper is a reflective review that highlights on the development of both physical infrastructure and organisational development of PHC, limited to the public sector which is the mainstream health provider. The evidences were collated through a literature search from governmental reports pertaining to Malaysia’s health-care sector, journal articles as well as grey literature. As there were several reforms that have taken place, the review was divided into five phases, from early conception of PHC development in Malaysia to year 2017. For each phase of the reform, documents were analysed for evidences of planning, philosophy underlying the health system development, where available data on outcomes.

## Findings

### Progressive physical re-structuring and organisational evolution were central to continuing improvement of access and equity

#### Phase 1: Geographic expansion of physical facilities with standard norms for staff and materials

At independence from its colonial past, Malaysia inherited a health-care system that was based in urban centres and commercial agricultural rubber estates while the majority of the population were rural dwellers who depended on unregulated practitioners of traditional medicine. During the post-independence period in the late 1950s until the late 1970s, the goal was to reduce the urban–rural health divide by establishing a Rural Health Service (RHS). The physical infrastructure of the RHS was a network of rural facility complexes. Each complex consisted of a ‘main health centre’, 4 health sub-centres and 20 ‘midwives’ clinics cum quarters’ (MCQs) and was intended to serve a rural population of 50 000. Each complex would have prescribed norms for staffing consisting of a doctor (known and as a ‘medical and health officer’ to be distinguished from ‘medical officer’ counterparts who worked in hospitals and provided only curative services), dental officer, medical assistants, laboratory technicians, pharmacy assistants, nursing, midwifery and sanitation personnel, vehicles equipment and materials. And they were to provide a standard package of basic preventive and curative health care (Ismail, 1978; Jaafar et al., 2007). Maternal and child health (MCH) and rudimentary outpatient (first contact clinical care) services were important core components of the services that were provided.

For the MCH component, midwives identified pregnant women in the village community and encouraged them to come to the midwife quarters cum-clinic which was the nearest health facility. Subsequently, a well-defined screening and referral system identified and referred mothers and children who needed more intense or complex supervision to progressively higher levels of care, namely the Public Health Nurse at the health sub-centre or the doctor at the main health centre. A key feature of the physical facilities was that the ‘Midwife Clinic Cum Quarters’ situated within the rural village. It had space for a simple clinic with attached living accommodation that was provided free to the midwife and her family. The MCQ was equipped with water and electricity and midwives were provided bicycles (which in later years were replaced by motorcycles). This living arrangement reduced absenteeism and ensured the availability of the midwife almost any time of the day (Munabi-Babigumira et al., 2017). Similarly, in the larger villages or small towns, housing was also constructed near the health sub-centre and the main health centre to cater for staff who provided ‘on-call services’ to the population.

The main health centres were designed to have two wings namely the MCH unit and the outpatient unit. This separation of function recognised the need or cultural acceptability for the conservative rural population who regarded pregnancy as highly personal. Women needed privacy from the outpatient clinics. MCH staff were able to gradually develop the trust of the female population and ante-natal, post-natal and child health attendances increased rapidly.

The shortage of doctors and dentists meant that the bulk of services were provided by allied health personnel, while the doctors and dentists visited on a rotational basis. However, population growth outstripped construction capacity. By 1975, only half of the expected expansion had been achieved with a typical three-tier network serving an average of 112 900 rural populations instead of the planned 50 000, with the average rural midwife serving 4390 populations instead of the planned 2000 (Noordin, 1978).

##### Reorganisation of the physical configuration of rural services to improve access

The then Director General of Health Malaysia, Dr Raja A. Noordin was an active contributor to the Alma Ata conference and provided the leadership for infusing the concepts central to Health for All into the health system. The Alma Ata Declaration provided an impetus to review the status of equitable access and institute strategies to accelerate progress. Recognising the construction of new physical facilities, each supplied with a standard norm of human resources and equipment was too slow to process, the three-tier system was converted to two tiers whereby existing sub-centres were upgraded to health centres, and midwives’ clinics were converted into multipurpose community clinics that would provide basic curative services in addition to the ongoing MCH care. Massive recruitment and retraining converted midwives into community nurses, and additional living accommodation was constructed adjacent to the multipurpose clinics to cater for new staffing norms with two community nurses per multipurpose clinic. The aim was to have coverage of 20 000 populations by each cluster.

##### Communities at the grassroots level contributed actively to the development of the health system

Another major contribution of Raja Noordin from Alma-Ata was the spur of mobilisation and active participation of the community in the development of the Malaysian health system. Initially termed a ‘community participation’, the concept was partnership between health services and community volunteers to improve health of communities. During the 1970s–1980s, efforts focussed on nutrition and sanitation (Noordin, 1978). Driven by the Gerakan Pembaharuan 1972 (Operation Research) focusing on the underserved population at the grassroots level, the community was motivated and guided to play a leading and complementing role in addressing the health agenda. The fixed facility network was supplemented by mobile health teams who collaborated with community volunteers (community health movements) on specific projects to improve nutrition and sanitation.

An intersectoral approach to improve education, health and living standards using the platform of the Applied Food and Nutrition Programme that had been introduced in 1969 was leveraged (Ismail, 1978). Outreach and community-based volunteers namely the Village Health Volunteers in Sarawak since the early 1960s, the Village Health Friends in Sabah since 1970s have been created and are still active to date. The activities then needed a physical space within the village. In some communities, the home of the village headman provided this space, while in other villages multipurpose community centres constructed by the ongoing rural development programme provided the physical facility which was shared by several implementation agencies for community-based activities in the rural areas.

The MCH was the major beneficiary of the early phase of PHC development. With the pervasive presence of the midwives and community nurses, the antenatal attendances improved each year. See Table 1 on maternal health coverage and the increasing trends of the average antenatal visits.

**Table 1.** Maternal health coverage in Malaysia, selected years 1990–2014

	1990	2000	2010	2011	2012	2013	2014
Estimated number of pregnant mothers	676 382	691 664	587 479	565 072	580 536	592 489	592 489
Antenatal coverage	528 029, 78.1%	517 138, 74.8%	483 136, 82.2%	550 104, 97.3%	560 323, 96.5%	580 819, 98.0%	575 604, 97.2%
Average ante-natal visits per mother	6.6	8.5	10.4	9.8	10.0	9.9	10.6

*Source*: MOH Annual Report 2014.

The improvements were accompanied with impressive decline of the maternal mortality ratio during the 50-year period, 1957–2013, from 530 deaths (Hematram, 2006) to 23.2 deaths per 100 000 live births. Infant mortality decreased by over 90% during the period 1957–2013, from 75.5 deaths to 6.5 deaths per 1000 live births (MOH, 2015).

#### Phase 2: Reorganisation and expansion of scope of PHC services (1986 onwards)

The thrust of the next phase of development was expansion of the scope of services provided as primary care level. The Eighth Malaysia Plan (1986–1990) introduced a new policy to extend PHC from just focusing on MCH to include the adolescent, adult and elderly thereby providing preventive services with seamless transition throughout life (Aniza et al., 2008). The practice then, services were delivered through dedicated clinic sessions. It soon became apparent that organisational change was needed to cope with demand. Sessional clinics, for example, dedicated maternal, child and family planning sessions conducted on separate days were integrated. During every integrated session, families were encouraged to make family appointments and all family members who came to the clinic together were given age- and condition-appropriate care. This enabled family to avoid multiple visits for different family members. New screening programme such as congenital hypothyroidism, autism, thalassaemia, wellness screening for adolescent, adult men and women and elderly, were some of the services that became available to the family.

##### New partnership with communities progressed

With the growing expansion of health infrastructure, several initiatives aimed at encouraging active community involvement to enhance and supplement the programmes offered though health clinic facilities. In 1990, the Health Clinic Advisory Panel in Peninsula Malaysia was created to support this agenda. They were made up of community volunteers appointed and ‘adopted’ by the nearest health clinics. Basic trainings were provided and collaborated with health staff, to develop health activities and targets for their communities (Pejabat Kesihatan Daerah Kota Tinggi, 2018). They were encouraged and given freedom to innovate suitable and acceptable community interventions and participate in delivering them. Activities ranged from MCH services, adolescent reproductive health, elderly care and control of diseases such as filariasis, malaria, tuberculosis and HIV. With the increasing threats of non-communicable diseases (NCDs), in 2013 another new volunteer initiative was launched known as KOSPEN which is an acronym for Healthy Community Healthy Nation. The main activities of the KOSPEN volunteers were to improve awareness of the community on NCDs and their risk factors, focusing on eating healthy, active lifestyle, no smoking, weight management and health screening. By 2014, more than 12 800 volunteers in 2014 had been recruited. An evaluation study in 2017 showed that KOSPEN volunteers had good knowledge in implementing the desired programme (Sondaram et al., 2017). However, there were misconceptions among the KOSPEN volunteers regarding their functions and roles and efforts are ongoing to address this issue.

#### Phase 3: Rationalisation of services: transfer of ambulatory services from hospitals to PHC clinics and process for greater integration (1994 onwards)

**Figure 1.**
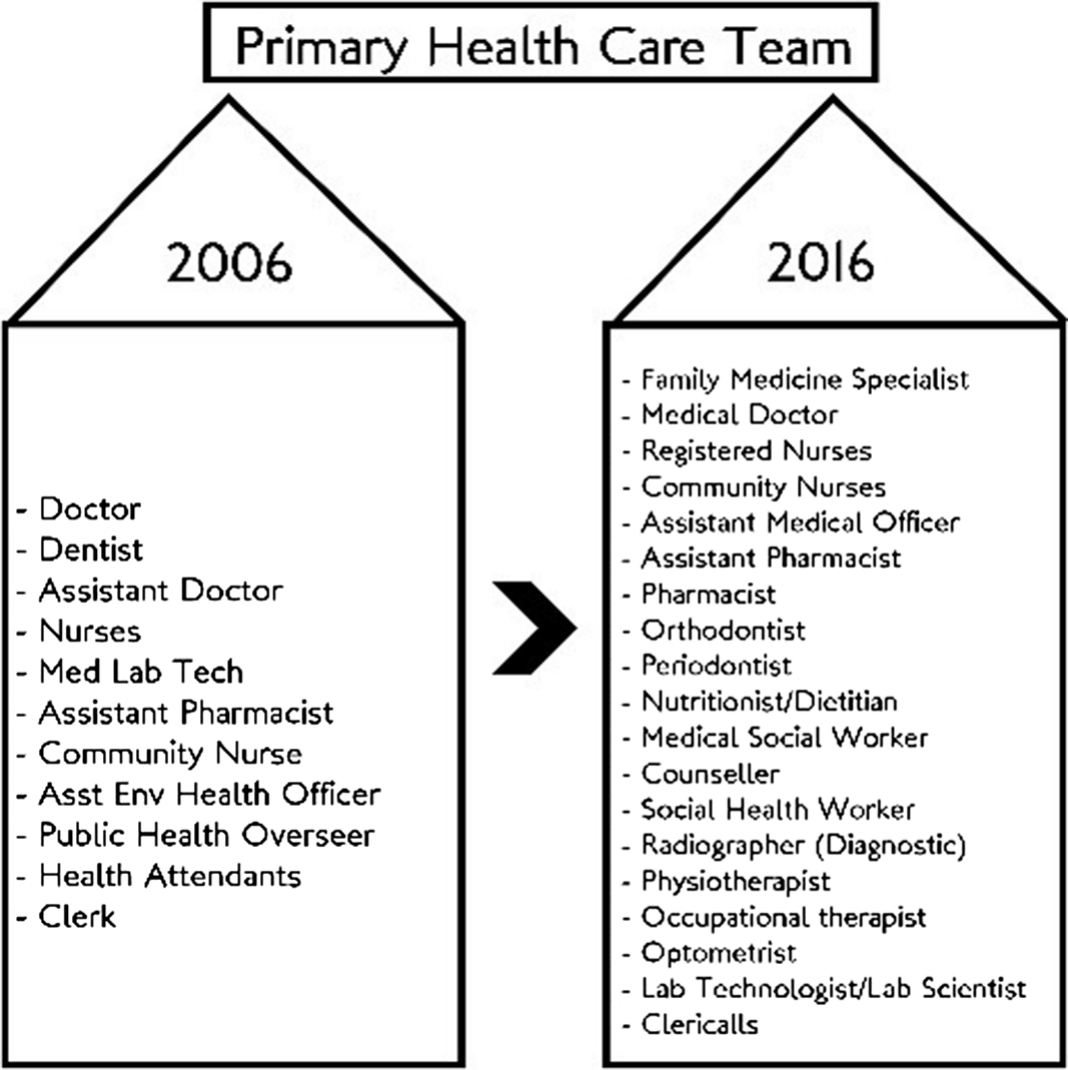
PHC team.

**Figure 2.**
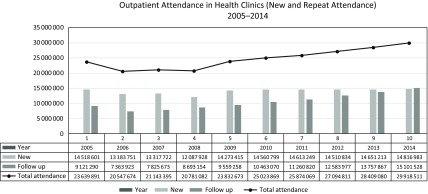
Outpatient attendances in health clinics (new and repeat) 2005–2014.

*Source*: MOH Annual Report 2014.

Historically outpatient (ambulatory) services that provided clinical (curative) services to walk-in patients were the domain of hospitals services (Sulvetta, 1992). In 1992, the MOH adopted a policy to transfer public sector outpatient services to the PHC (MOH, 1998). There were two reasons for this decision. First, in accordance with the principles of primary care in the Alma Ata Declaration, it would enable the public to obtain care not only for their current symptoms but also have relevant promotive, preventive and rehabilitative care through a single package of services at the health clinic level. Second, it enabled more effective and efficient use of hospital staff and ward services by allowing hospitals to focus on secondary and tertiary care. During the period 1994–2000, the transfer of services was carried out in stages. It involved infrastructure, management, and human and budgetary resources. The clinics were gradually equipped with non-specialist radiological services manned by radiographer, a wide range of laboratory services manned by scientist and medical laboratory technicians. The dispensaries were upgraded into full pharmacy staffed by pharmacists. See Figure 1 for the expanded PHC Team. Although the exercise was planned to be completed by 1999, by 2000, only about 82.3% of outpatient services had been transferred successfully (MOH, 2000). The challenges were the need to accommodate more number of outpatients’ attendances transferred from the hospitals, with the closure of almost all the 124 hospital outpatient departments during this period. Cases that are on follow-up at the hospital outpatients and specialist clinics were also gradually passed-over to the health clinic to follow through. Figure 2 shows the increasing trends of new cases each year attending the health clinics and together with repeat cases have increased from 2 to 3 million over 10 years.

**Figure 3.**
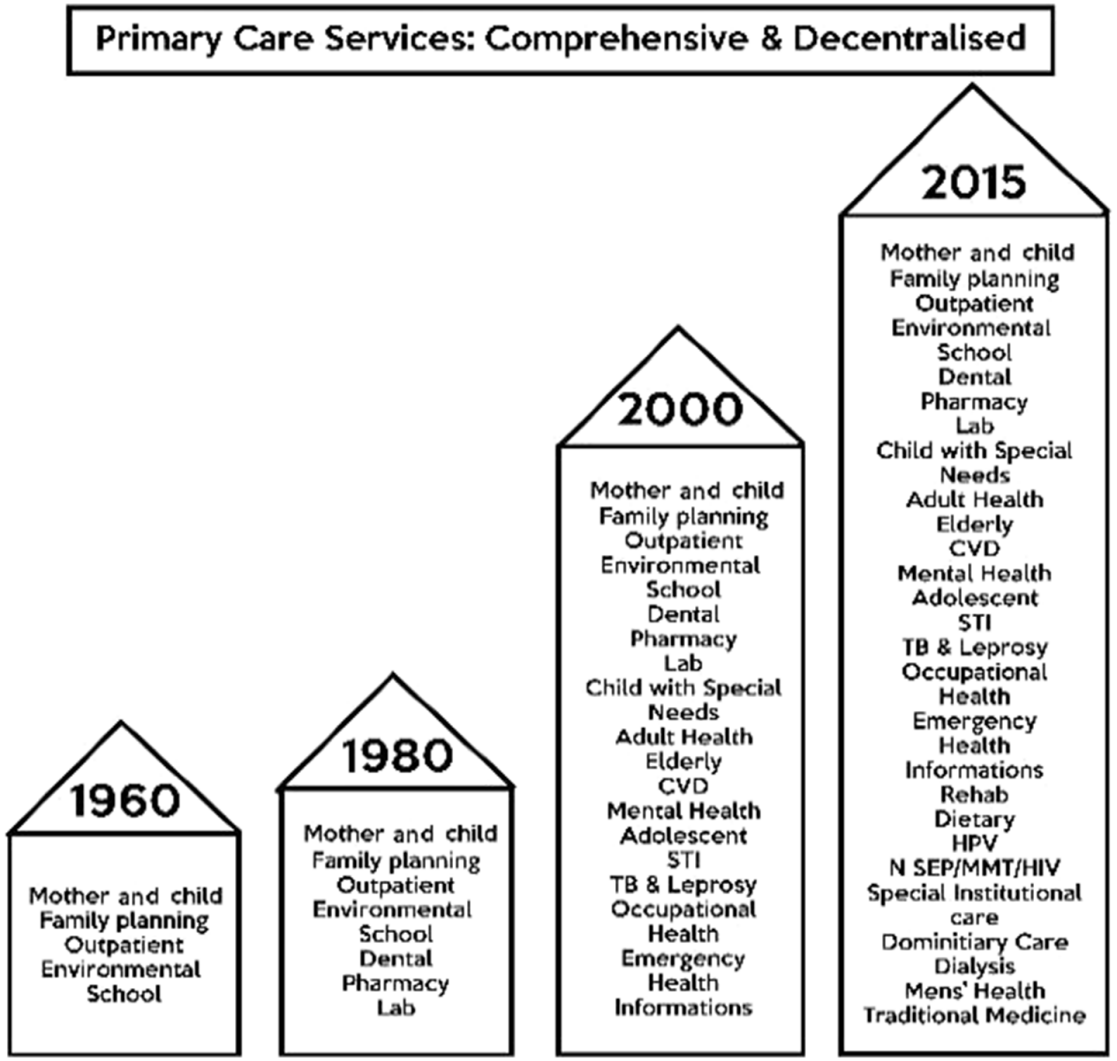
Increasing scope of services with introduction of new activities.

*Source*: Family Health Development Division 2015

Although simple and appealing in concept, this move produced several challenges that triggered further development of the PHC approach. The objective of providing comprehensive PHC came to fruition when plethora of programmes, between year 1986 and 2006, were created to be delivered through the health clinics. The new setting encouraged many hospital specialist disciplines to propose ‘downloading’ some of the hospital services to the health clinics that would be closer to home and improve access particularly for follow-up cases. The list of gradual increase of services is illustrated in Figure 3.

This euphoria however did not come without challenges. Services were transferred without parallel transfer of budget and human resources. The activities were driven either by global health drives or by local needs and demands. They were owned and managed by different units and divisions in the Ministry. The different heads dictating the implementation process, supplies and performance targets work in silos, plummeting the various programmes onto the ground staff to execute on the integration with little capacity. The enthusiasm for integration unfortunately was not accompanied with adequate collaboration and consultations and there was insufficient recognition of the changes needed in administrative processes, integration of personnel, budget and finance.

**Figure 4 and Box 1.**
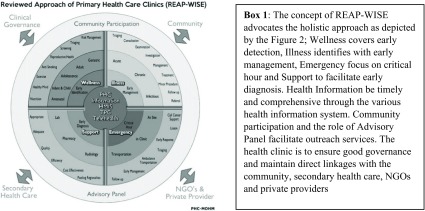
The reviewed approach of PHC and WISE components.

Hence, while integration of programmes and services, both horizontal and vertical, was the buzz word for more efficient and effective delivery of services through PHC, the haphazard implementation called for a need to reorganise the delivery system. After several consultations with all stakeholders, the ‘Reviewed Approach of Primary Health Care’ (REAP) was created (Jaafar, 2008). It has five components, namely wellness, illness, emergency, support and PHC information (see Figure 4 and Box 1).

Driven by the telehealth initiatives (Suleiman, 2001), the MOH attempted to reach the remote population through the introduction of teleprimary care (TPC) a home-grown ICT project that builds on the development of a clinical information system that allows for teleconsultation. The initial pilot that involves three states has allowed the solution to demonstrate how rural population can access to specialist consultation through TPC. A study done by Ringga showed how the Paediatric Department in Sibu Hospital in Sarawak started a video conferencing clinic that allowed patients in Belaga Health Clinic to have face-to-face consultations with paediatricians in Sibu Hospital (Ringga et al., 2011). Today the TPC has expanded to cover more areas in eight states with additional modules to cover dental services which are now upgraded to the Teleprimary Care and Oral Health Clinical Information System (Family Health Development Division, 2018).

#### Phase 4: Improving better quality and access to more comprehensive care at the primary care level (2000)

The year 2000 was a landmark period for PHC with the introduction of family medicine specialist (FMS) services in primary care (Aniza et al., 2008). Prior to this, it was the norm for primary care to be provided by doctors who had basic medical degrees but no postgraduate qualification. Patients with more complex conditions were referred to hospital-based specialists who had postgraduate qualifications in a relevant discipline. The risk in this system was that care for an individual patient would be fragmented as different specialists only dealt with conditions within their speciality, and no one had the responsibility for the patient as a whole. Specialisation in Family Medicine was introduced as a postgraduate specialist training for doctors in primary care to enable them to provide a more holistic clinical and preventive care at the primary care level. The REAP-WISE, an acronym for “Reviewed approach in primary care (REAP) – Wellness, Illness, Support, Emergency (WISE)”, see Figure 4 and Box 1, is an approach that blended well with the availability of the FMS providing the ‘person-care services’: the wellness, illness, emergency and support requirements, a departure from discipline-based or project-based approach (MOH, 2008).

This policy created a big change in the way the health clinics delivered services. The new approach required parallel development and enhancement of the PHC physical facilities infrastructure (Jaafar et al., 2007). Existing clinic infrastructure was reviewed and clinic building layouts for the future health clinics were revised to cater for a more equitable and efficient process and procedures of the health clinic PHC delivery.

**Figure 5.**
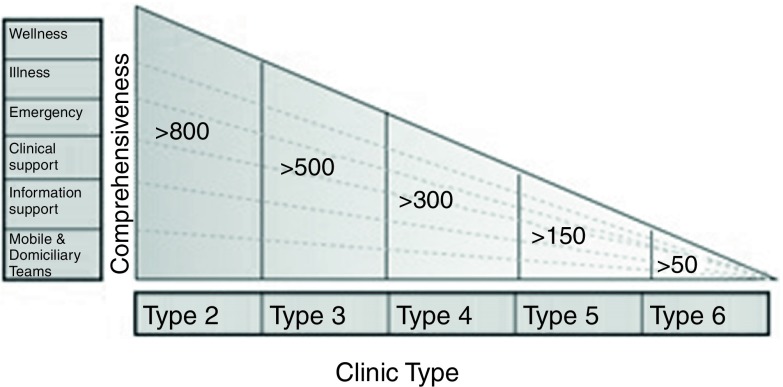
Type of clinics and the corresponding scope of services.

New clinic standard designs under the Medical and Design Brief 2015 were developed to cater for the need of new equipment, technologies, spaces and new patient and information flow. Different types of clinic designs (see Table 2 and Figure 5) were developed to meet the population and patient volume in different settings, with each type offering a comprehensive range of wellness, illness, emergency, support, information and mobile services.

**Table 2.** Health clinic type with estimated coverage area population and estimated daily patients’ attendances

Types of health clinics	Scope of services provided	Estimated population in coverage area based on (catchment population)	Estimated patients’ attendances daily
KK2	Outpatient, maternal and child, rehabilitation, x-ray, laboratory, dental health	>50 000	>800
KK3	Outpatient, maternal and child, rehabilitation, x-ray, laboratory, dental health	>30 000 – 50 000	>500–800
KK4	Outpatient, maternal and child, rehabilitation, x-ray, laboratory, dental health	>2 000–30 000	>300–500
KK5	Outpatient, maternal and child, x-ray, laboratory, dental health	>10 000–20 000	>150–300
KK6	Outpatient, maternal and child, laboratory, dental health, alternative birthing centre and observation beds (optional)	>5000–10 000	>100–150
KK7	Outpatient, maternal and child, laboratory, alternative birthing centre and observation beds (optional)	<5000	>50–100

*Source*: Planning Division and Family Health Development Division, MOH 2015.

Although the numbers of health clinics and community clinics increase over the last 40 years, the targeted ratio of health clinics (then Main Health Centre) and community clinics (then MCQ) at 15 000–20 000 and 3000–4000, respectively, to the population were not achieved (see Table 3). Budget is the major constraints. As stop-gap measures, smaller static clinics (Klinik 1 Malaysia) and mobile and outreach clinics were made available.

**Table 3.** Ratio of health facilities to population, year 1975 compared with year 2015

Types of clinics^1^	Year 1975^1^	Population to clinic^1^	Types of clinics^2^	Year 2015^2^	Population to clinic
Main health centre	62	112 900	Health clinics	991	31 988^*^
Sub-centre	239	23 250		–	–
MCQ	1293	4390	Community clinics	1921	16 502^*^
Total population		7 000 000			31 700 000^3^

*Source*: ^1^Noordin RAA 1978,

2 Family Health Development Division, MOH, 2015.

3 Department of Statistics 2017.

* Author’s calculation population divide by clinics.

Although the health clinics were short of the norm, the population continues to enjoy access to health services from the equally growing numbers of private general practitioner’s clinic. In 1986, the NHMS1 found that 74% of the population lived within 3 km of a modern static facility (Institute of Public Health, 1986). In 1996, the NHMS2 showed that within 3 km, 81% of the population lived nearest to a private clinic (Institute of Public Health, 1996). In 2006, the NHMS3 findings showed that the average distance from a private or a public clinic was almost equal 4.5 km from the household sampled (Institute of Public Health, 2008). The NHMS 2015 under Healthcare Demand Study found that the mean distance between government outpatient health-care facilities and respondents’ home was 9.8 km while the mean distance between private outpatient health-care facilities and respondents’ home was 9.3 km (Institute for Public Health, 2018). The spread and availability of public have caught well and allow the population almost equal access to a private or a public clinic. The scope in the public clinics has, however, expanded as has been described in Figure 3 as compared to the Private Clinics which are very much outpatient in approach.

#### Phase 5: Beyond equity to personalised care and universal health coverage (2013)

##### Family doctor and concept and personalised care

**Figure 6.**
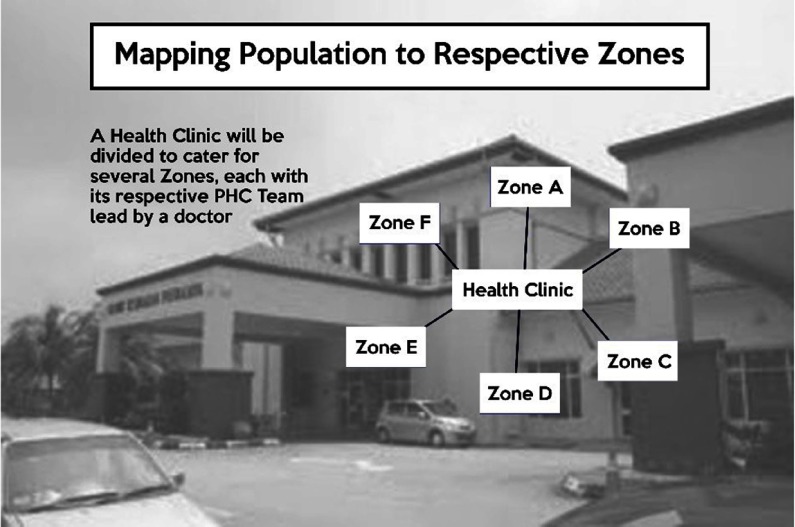
Mapping population to respective zones.

The number of medical doctors serving in primary care in the public clinics continues to increase. Concurrently, as the concept of Universal Health Coverage was being explored, it has succeeded with the introduction of the Family Doctor Concept in 2013 leveraging on the availability of these doctors. Every citizen of Malaysia can gain access to the public sector clinics for a nominal payment, if not for free. Each health clinic covers a population ranging from 10 000 in rural settings to as many as 100 000 in urban settings. A study that was conducted in 2016 (Safurah J, 2018) described the phased implementation by clustering the population in the catchment area of a clinic into zones and registering each zone with a PHC team lead by a medical doctor (see Figure 6). The concept aims to provide a personalised and continuity of care by establishing patient–doctor relationship between each family with a particular doctor covering primary level curative care, together with the promotive, preventive and rehabilitative elements of the PHC services.

##### Challenges on the road to equity and access

The government budget for public sector primary care services is allocated on the assumption that it is targeted to the lower socioeconomic strata and the poor. However, utilisation by the non-poor is neither triaged nor prohibited. Every Malaysian has the right to access PHC in Malaysia. With ever-increasing attendances, the allocated ringgit has to be stretched to provide the most efficient delivery possible.

On a favourable note, the Malaysia Healthcare Demand Analysis study (Ravi P. Rannan-Eliya, 2013) suggested that utilisation of health services ‘has reached comparable levels of OECD Countries’. There is adequate protection of financial risk for the poor and simulation model that was performed revealed a fair distribution of resources between the rich and poor households.

Lim et al. (2017), however, in their paper ‘Chasm in primary care provision in a universal health system: findings from a nationally representative survey of health facilities in Malaysia’, shared that the distribution of health services and resources within the public sector was, however, unequal with a higher propensity towards urban clinics. Offering a proposition in order to improve further access and coverage to the population efforts if taken, should be directed towards expansion of facilities in rural areas.

There will always remain a continuing dilemma between cost-effectiveness, access and equity. Services such as the flying doctor service, the birthing centre in the health clinics, are amongst services that have high capex but serving low volume of patients although much desired for the population in remote location. It is inevitable to focus on these few interventions to address equity. However, more efforts are required to look at opportunity costs of alternative activities in altering the behaviour of individuals or institutions. The telehealth and TPC initiatives with the slogan ‘Care Close to Home’ are potential sustainable efforts that may offer innovative ways to improve access to deliver care to the population.

## Conclusion

Malaysia has made significant strides over the last 40 years in reducing the urban–rural differentials towards accessibly to quality health care. Sivasampu et al. (2015) in their study have shown that the urban and rural patients do not perceive differences in access to health-care services. Impressive health indicators have also been a testimonial of the appropriate interventions that have been instituted. Despite the limited public funding, the country has generated significant delivery efforts in providing the timely policies, appropriate infrastructure with relevant human resource development, targeting equitable access to PHC services for its population, ‘leaving no one behind’, whether they are in rural or urban, remote or in the heart of the city.
