# 40 years of Alma Ata Malaysia: Targeting equitable access through organisational and physical adaptations in the delivery of public sector primary care – CORRIGENDUM

**DOI:** 10.1017/S1463423620000146

**Published:** 2020-05-21

**Authors:** Fariza Fadzil, Safurah Jaafar, Rohana Ismail

In the original paper, the author affiliation listed incorrectly. The correct list is as follows:

Fariza Fadzil^1^, Safurah Jaafar^2^ and Rohana Ismail^3^

^1^Doctor of Public Health and Master of Public Health, Senior Principal Assistant Director, Primary Health Infrastructure Development Sector, Primary Healthcare Section Family Health Development Division, Ministry of Health Malaysia, Putrajaya, Malaysia; ^2^Master of Public Health, Master of Business Administration, Former Director Family Health Development Division, Ministry of Health Malaysia, Currently Professor Community Medicine, International Medical University Malaysia, Kuala Lumper, Malaysia and ^3^Master of Public Health, Senior Principal Assistant Director, Primary Health Infrastructure Development Sector, Primary Healthcare Section Family Health Development Division, Ministry of Health Malaysia, Putrajaya, Malaysia

The authors would like to apologise for this mistake. This has been corrected in the online PDF version.
